# Iron deficiency in the elderly population, revisited in the hepcidin era

**DOI:** 10.3389/fphar.2014.00083

**Published:** 2014-04-23

**Authors:** Fabiana Busti, Natascia Campostrini, Nicola Martinelli, Domenico Girelli

**Affiliations:** Department of Medicine, University of VeronaVerona, Italy

**Keywords:** iron deficiency, anemia, elderly, hepcidin, ferritin

## Abstract

Iron deficiency (ID) is relatively common among the elderly population, contributing substantially to the high prevalence of anemia observed in the last decades of life, which in turn has important implications both on quality of life and on survival. In elderly subjects, ID is often multifactorial, i.e., due to multiple concurring causes, including inadequate dietary intake or absorption, occult bleeding, medications. Moreover, because of the typical multimorbidity of aged people, other conditions leading to anemia frequently coexist and make diagnosis of ID particularly challenging. Treatment of ID is also problematic in elderly, since response to oral iron is often slow, with a substantial fraction of patients showing refractoriness and requiring cumbersome intravenous administration. In the last decade, the discovery of the iron regulatory hormone hepcidin has revolutionized our understanding of iron pathophysiology. In this review, we revisit ID among elderly people in the light of the impressive recent advances on knowledge of iron regulation, and discuss how hepcidin may help in diagnosis and treatment of this common clinical condition.

## ANEMIA IN ELDERLY, PREVALENCE, AND DEFINITION

Anemia is a common, multi-factorial condition in elderly. Indeed, the prevalence of anemia increases with age, representing an important health problem among older individuals. Large studies on community-dwelling older adults from the United States and Europe have reported prevalence rates for anemia ranging from 8 to 25% (Patel, 2008). One of the largest population survey, i.e., the third US National Health and Nutrition Examination Survey (NHANES III), indicated that 10.2% of women and 11% of men >65 years of age were anemic (Guralnik et al., 2004). These fractions rose to 26.1 and to 20.1% in subjects older than 85 years old, in males and females, respectively (Guralnik et al., 2004).

There is some debate on which hemoglobin (Hb) threshold should be used to define anemia in the general population and particularly in elderly individuals (Beutler and Waalen, 2006). In many studies, anemia has been defined according to the World Health Organization (WHO) criteria (Blanc et al., 1968) as a Hb level <13 g/dL in men and <12 g/dL in women, respectively. However, these criteria have been criticized since they were based on statistical distributions (i.e., equivalent to two standard deviations below the mean) in reference samples that did not include individuals >65 years of age, making unfeasible their application to older individuals (Mindell et al., 2013). Since Hb values in apparently healthy elderly individuals are generally lower than those in younger adults and the differences between males and females tend to disappear with aging (Patel, 2008), a Hb value < 12 g/dL is now commonly considered indicative of anemia in elderly of both sexes (Izaks et al., 1999; Andrès et al., 2013).

Anemia in elderly is typically hyporegenerative and relatively mild, with Hb levels near 10–11 g/dL in most subjects (Guralnik et al., 2004). Nevertheless, it is associated with a variety of adverse outcomes, including longer hospitalization, disability, and increased mortality risk (Chaves et al., 2004; Zakai et al., 2005; Culleton et al., 2006; Denny et al., 2006; Penninx et al., 2006; den Elzen et al., 2009; Price et al., 2011). Moreover, it also significantly impacts on the quality of life, being associated with fatigue, cognitive dysfunction, depression, decreased muscle strength, falls, and “frailty,” even when Hb levels are merely low-normal (Woodman et al., 2005; Eisenstaedt et al., 2006).

Approximately, one-third of the cases of anemia in elderly can be ascribed to a chronic disease (inflammation and chronic kidney diseases), and one-third is due to nutrient deficiencies (folate, B12, and iron). Iron deficiency (ID), alone or in combination with deficiency of other nutrients, accounts for more than one-half of this group. The last third remains “unexplained” (Guralnik et al., 2004). Noteworthy, a significant proportion of elderly anemic patients (30–50%) is presumed to have multiple causes of anemia (Petrosyan et al., 2012). Since elderly patients are typically affected by several different pathologic conditions (multimorbidity), and are commonly taking a long list of medications, the precise etiology of anemia is often difficult to determine in a given individual (Andrès et al., 2013), and sometimes remains “unexplained” despite extensive investigation (Guralnik et al., 2004). Thinking in terms of multimorbidity is a key to understanding, diagnosis, and treatment of anemia in the elderly.

## IRON DEFICIENCY IN ELDERLY

According to the WHO, ID is by far the most common and widespread nutritional disorder worldwide (http://www.who.int/nutrition/topics/ida/en/), with estimated one billion people affected, thus constituting a public health condition of epidemic proportions. Besides the large number of children and young women affected in developing countries, ID is the only nutrient deficiency that is also significantly prevalent in industrialized countries [World Health Organization (WHO), 2001; Hershko and Camaschella, 2013], where an additional category at risk is represented by elderly people (Guyatt et al., 1990).

Iron deficiency syndromes include a range of different conditions (Goodnough, 2012). “*Absolute*” ID is defined by the lack of storage iron (Cook, 2005; Fairweather-Tait et al., 2013). In physiological conditions, the total body iron amount (near 3–4 g) is maintained by a fine balance between three distinct factors: body requirements, iron supply (depending on dietary iron intake and duodenal absorption), and blood losses. While an increased iron demand is the main cause of ID in children and fertile females, insufficient dietary iron intake, gastrointestinal (GI) malabsorption and/or increased blood losses are the most common causes of ID in older individuals (see below).

At variance with “absolute” ID, many disorders are characterized by the so-called “*functional*” or “*relative*” ID, defined as the occurrence of iron-restricted erythropoiesis in presence of normal or even increased amounts of body iron stores. This phenomenon is often related to an impaired iron trafficking (i.e., block of iron release from macrophages and hepatocytes, typically during inflammatory diseases) or to increased/ineffective/stimulated erythropoiesis, with iron demand exceeding the supply (i.e., during hemoglobinopathies, chronic hemolytic anemias or treatment with erythropoiesis stimulating agents). Since the focus of this article is on etiology, diagnosis, and management of the absolute ID in elderly, the readers are referred to others excellent reviews for details on the functional ID syndromes (Goodnough et al., 2010, Goodnough, 2012; Auerbach et al., 2013a).

Whatever the mechanism, both absolute and functional ID reduce iron availability to erythroid precursors, with the development of an iron-restricted erythropoiesis, and finally of anemia. In particular, two ID stages can be distinguished: (a) initial, characterized by reduced transferrin saturation but without anemia; and (b) advanced, when microcytic, hypochromic iron-deficiency anemia (IDA) becomes evident.

**FIGURE 1:**
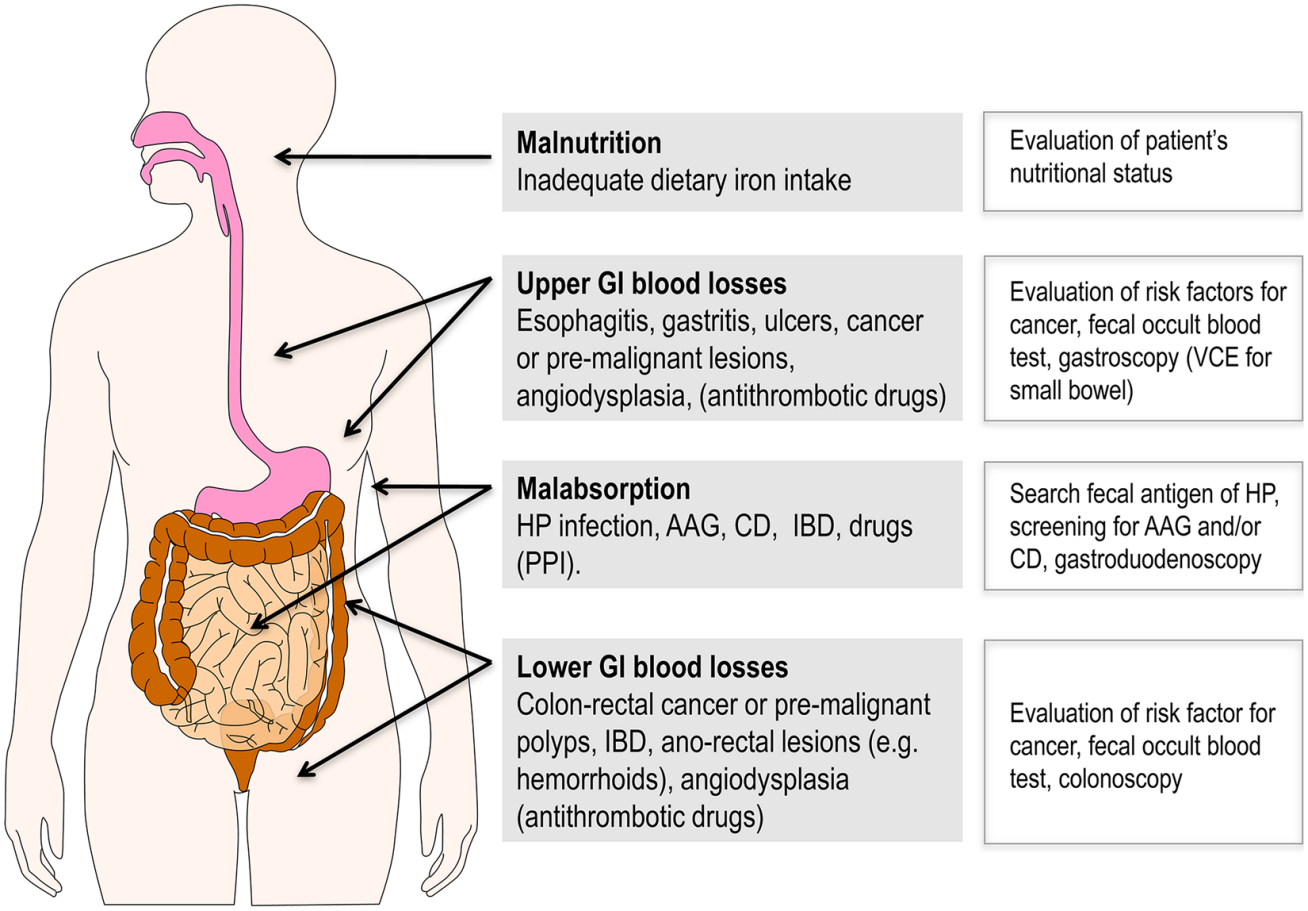
**Gastrointestinal diseases representing the most frequent causes of ID and IDA in elderly patients**. Of note, more than one of these conditions not infrequently coexist in a given individual. Bleeding is often favored by antithrombotic drugs for treatment of cardiovascular diseases that are highly prevalent in this age group. Suggested diagnostic tools are reported on the right side. AAG, autoimmune atrophic gastritis; CD, celiac disease; GI, gastrointestinal; HP, *Helicobacter pylori*; IBD, inflammatory bowel disease; PPI, proton pump inhibitors; VCE, video capsule endoscopy.

In elderly, ID and IDA are nearly always due to chronic GI diseases, which in turn lead to iron loss and malabsorption not infrequently occurring in combination at individual level (**Figure 1**). Indeed, the most frequent cause is represented by *chronic upper and lower GI blood losses*, because of esophagitis, gastritis, peptic ulcer, colon cancer or pre-malignant polyps, inflammatory bowel disease, or angiodysplasia (Eisenstaedt et al., 2006). The prevalence of most of these conditions increases with age, which is particularly true for neoplastic lesions (Eddy, 1990) and angiodysplasia (Sami et al., 2014). Remarkably, GI bleeding is typically increased by concomitant assumption of medications for conditions highly prevalent in elderly individuals, such as non-steroidal anti-inflammatory drugs for osteoarthritis, and antithrombotic therapies for cardiovascular disease, especially for atrial fibrillation.

*Iron malabsorption* is also relatively frequent in the elderly. Indeed, further conditions whose prevalence typically increases with age are represented by *Helicobacter pylori* (HP) infection (Pounder and Ng, 1995) and atrophic gastritis. Of note, although, for a long time, celiac disease (CD) has been primarily considered an enteropathy of childhood and young adults, a number of epidemiological studies have reported an increased detection rate in older subjects, with up to one third of newly diagnosed patients being older than 65 years (Patel et al., 2005; Rashtak and Murray, 2009; Vilppula et al., 2009). In this age group, multifactorial anemia is the most frequent clinical presentation (Harper et al., 2007), with micronutrients deficiency (particularly ID) being the leading cause. For poorly understood reasons, the classical triad of malabsorptive symptoms including diarrhea, weight loss and abdominal pain is less common in elderly (Freeman, 2008), making the diagnosis frequently overlooked in this age category. Another factor that could theoretically contribute to iron malabsorption in elderly patients is represented by the frequent long-term use of proton pump inhibitors (PPI), being gastric acid essential for optimal intestinal absorption of the element (Ganz, 2013). However, only few reports have specifically addressed this issue, which remains controversial (Reimer, 2013).

Typically, all the above-mentioned conditions impairing iron absorption share a clinical phenotype of refractoriness to oral iron therapy, recently named “acquired IRIDA” (iron refractory ID anemia; for a review see Hershko and Camaschella, 2013). These conditions should be always considered in elderly subjects with IDA and no evidence of GI blood loss.

Finally, *malnutrition* is an obvious contributing factor to ID in elderly. However, since iron requirement (1–2 mg/day) only corresponds to near 10% of the average daily iron intake, malnutrition is rarely sufficient *per se* to cause IDA, at least in industrialized countries. Nevertheless, evaluation of the patient’s nutritional status plays an important role in the diagnostic approach to anemia in the older adult.

## HEPCIDIN, THE KEY REGULATOR OF IRON HOMEOSTASIS

Hepcidin, a defensin-like hormone synthesized mainly by the liver, has been discovered in 2001 and recognized as the master regulator of iron metabolism (Ganz and Nemeth, 2011). The active form of hepcidin is a 25-amino acid peptide derived from an 84 amino acid precursor, but at least two others isoforms truncated at the N-terminus, i.e., hepcidin-20 and hepcidin-22, have been also identified in biological fluids (Castagna et al., 2010). The biological meaning of these isoforms is still unclear (Campostrini et al., 2012). Hepcidin acts by binding to its receptor, the transmembrane protein ferroportin, which currently represents the only known cellular iron exporter in mammals (De Domenico et al., 2011). In humans, ferroportin is mainly expressed in cells playing a key role in iron homeostasis, like duodenal enterocytes (absorption of dietary iron), in splenic and hepatic macrophages (recycling iron from erythrophagocytosis), and in hepatocytes (iron stores). The hepcidin-ferroportin binding induces the endocytosis and the lysosomal degradation of both molecules, resulting in decreased intestinal absorption and release of iron from recycling macrophages, both ultimately leading to reduction of plasma iron concentration (Ganz and Nemeth, 2011). Regulation of hepcidin synthesis is quite complex and includes a number of different pathways [for recent detailed reviews see Ganz (2013) and Meynard et al. (2014)]. ID and increased erythropoietic activity down-regulate hepcidin production, and suppressed or very low hormone concentrations are observed in IDA or anemias with high erythropoietic activity (Ganz et al., 2008). Although the nature of the suppressive signal is still unknown, there is some evidence that, at least in conditions of stimulated erythropoiesis, it could be represented by a circulating factor produced by the erythroid progenitors in the bone marrow (Kautz et al., 2013). On the other hand, hepcidin is strongly induced by inflammation (Nemeth et al., 2003, 2004), in particular by the pro-inflammatory cytokine interleukin-6 (IL-6), and it is responsible for iron-limited erythropoiesis in patients with acute and chronic inflammatory states (Ganz, 2003; McCranor et al., 2013). Nevertheless, recent studies in mouse models (Gardenghi et al., 2014; Kim et al., 2014) have suggested that the iron-restricted anemia induced by inflammation likely recognizes a more complex pathogenesis, only partially dependent on hepcidin.

Currently, two main methods are available for measuring hepcidin in blood and urine, immunoassays based on anti-hepcidin antibodies, and mass spectrometry (MS)-based assays (Castagna et al., 2010; Kroot et al., 2011). The latters are generally preferable, being able to distinguish the iron bioactive 25-mer isoform from other isoforms of uncertain significance, at variance with the incomplete specificity of available antibodies (Castagna et al., 2010; Kroot et al., 2011). Serum hepcidin shows well-defined age- and sex-specific variations at population level as illustrated below (Galesloot et al., 2011; Traglia et al., 2011). The measurement of hepcidin in biological fluids represents a promising tool in the diagnosis and management of conditions characterized by an altered iron homeostasis, including ID/IDA. However, a “gold standard” method available for daily clinical practice at reasonable costs is stills lacking.

## HEPCIDIN LEVELS IN ELDERLY

**FIGURE 2:**
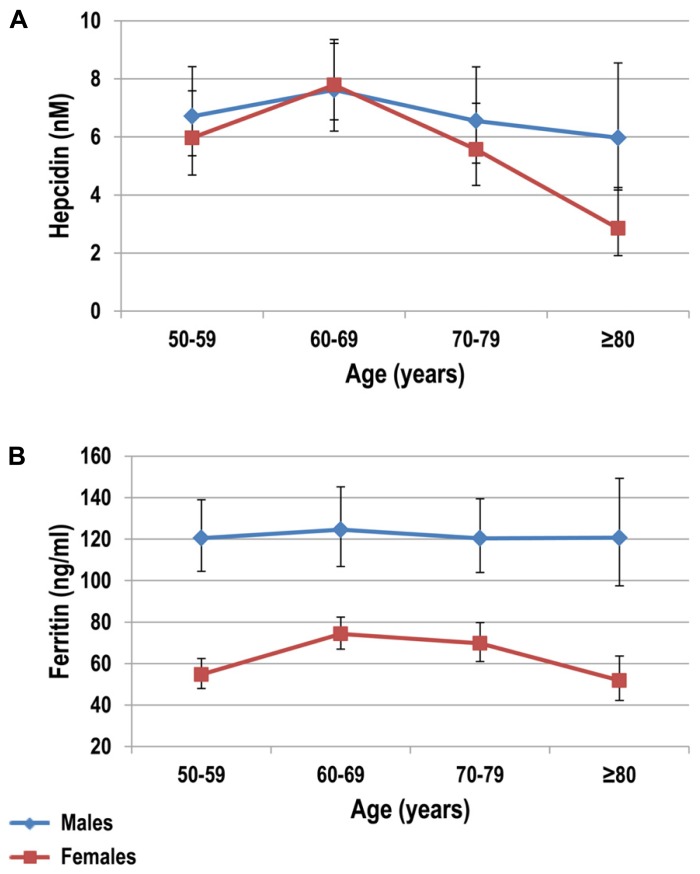
**Serum hepcidin (A) and ferritin (B) levels stratified by decades in healthy subjects older than 50 years**. Subjects were from the Val Borbera study, a large population survey including 1657 subjects. Adapted from Traglia et al. (2011).

The “unexplained” anemia of elderly has been linked to two putative mechanisms, namely a progressive resistance of bone marrow erythroid progenitors to erythropoietin (EPO), and a chronic subclinical pro-inflammatory state (Vanasse and Berliner, 2010). In this context, hepcidin could theoretically play a substantial role, considering its involvement both in inflammation and in the regulation of iron availability for erythropoiesis. Indeed, previous studies have showed a mild increase of inflammatory markers like tumor necrosis factor alpha (TNF-α) and IL-6, a major hepcidin inducer, in elderly subjects (Andrews, 2004; Ferrucci et al., 2005; Maggio et al., 2006). To date, only two studies, the Nijmegen biomedical studies (NBS; Galesloot et al., 2011), and the Val Borbera studies (VBS; Traglia et al., 2011), have investigated serum hepcidin levels at population level in apparently healthy subjects, including elderly groups. Both studies showed that before the menopause hepcidin levels in women are nearly 50% lower than in males of corresponding ages. After the menopause, hepcidin levels tend to be similar in both sexes, with a slight decrease in the eldest groups. This was evident in both sexes in the VBS (**Figure 2**), but only in males in the NBS. Although not specifically designed to study the anemia of elderly, these studies tended to exclude a sustained increase of hepcidin in elderly. Accordingly, two studies in elderly anemic patients have failed to detect increased hepcidin levels in urine (Ferrucci et al., 2010) and in serum (Waalen et al., 2011), and even a correlation between hepcidin and IL-6 or TNF-α (Ferrucci et al., 2005).

On the contrary, the Leiden 85-plus Study, a population-based prospective study in 85-year old subjects from Leiden (the Netherlands), showed that in this age group C-Reactive Protein (a proxy for IL-6) was a significant predictor of circulating hepcidin levels, which in turn were relatively higher in a small subgroup (*n* = 29) with unexplained anemia (den Elzen et al., 2013). Several reasons may account for these discrepancies on hepcidin levels in elderly anemic subjects, including the different laboratory methods and clinical settings (Goodnough and Schrier, 2014). Most of the data are retrospective, and properly designed, large-scale, studies are required before drawing definite conclusions. For the moment, the available evidence has generally downsized the initial hypothesis on hepcidin as a major determinant of the unexplained anemia of the elderly. This remains likely a complex condition due to the combination of several age-related changes, such as stem cell aging, low-grade chronic inflammation, subclinical impairment of kidney function, androgen insufficiency, and others still unknown (Guralnik et al., 2004; Makipour et al., 2008).

## DIAGNOSIS OF IDA IN ELDERLY

Several guidelines and recommendations have been proposed for the diagnosis of IDA in the general population (Cook, 2005; Goddard et al., 2011), but there is no consensus regarding the optimal approach for the diagnosis and management of IDA in elderly. Nevertheless, it is clear that, besides iron supplementation, the general principle of searching, and if possible treating, the underlying cause(s) should be pursued also in elderly patients (Andrès et al., 2013). Being GI diseases the most common causes of IDA in elderly (**Figure 1**), the diagnostic work-up should often, at least theoretically, include relatively invasive investigations, like endoscopic procedures. This is particularly true since, for example, IDA in elderly often herald the presence of an occult GI malignancy. Of course, old age *per se* is not a contraindication to such procedures, but a particular clinical skill is required in each individual and frail elderly patient to thoroughly evaluate the risk-benefit ratio as well as the prognostic implications.

Anyway, while the diagnostic work-up should be, whenever possible, comprehensive, some conditions deserve peculiar attention in the elderly patient presenting with IDA.

In our experience, a condition particularly challenging is represented by GI angiodysplasia, which in turn is a potentially treatable disease (Richter et al., 1984). Remarkably, bleeding in GI angiodysplasia is often discontinuous, with possible false negativity of occult fecal blood test. Moreover, although most of GI angiodysplasia lesions are localized in the colon (54–82% are in the cecum and in ascending colon), they can escape to a single endoscopy, or be localized in the small bowel, which is not routinely investigated. In this case, additional testing by video capsule endoscopy (VCE) is needed (Sami et al., 2014). Finally, a distinct feature of GI angiodysplasia is its frequent association with another relatively common condition in elderly, i.e., aortic stenosis, which occurs in near one third of patients (Batur et al., 2003). This association, known as Heyde’s syndrome, is notably characterized by a coagulopathy, i.e., acquired von Willebrand disease (Vincentelli et al., 2003), due to shear-stress mediated consumption of high molecular weight multimers of the von Willebrand factor (Loscalzo, 2012). Being the latters the most hemostatically competent form of von Willebrand factor, this favors a vicious circle that aggravates the bleeding from GI angiodysplasia and the consequent IDA.

From a laboratory point of view, an accurate diagnosis of IDA in elderly is also challenging because of the high prevalence of concomitant chronic diseases that complicate the interpretation of traditional biomarkers. The red blood cells mean corpuscular volume (MCV) is often a starting index in the evaluation of a patient with anemia, being typically reduced in IDA. However, MCV reduction in elderly is often lacking in early stages and/or blunted by other concomitant nutritional deficiencies, such as those of folic acid or vitamin B12. Similarly, the other common laboratory markers of “absolute” ID, i.e., low serum ferritin and transferrin saturation, and raised transferrin, have a low sensitivity in elderly (Fairweather-Tait et al., 2013). For example, the classical cut-off value of serum ferritin ≤12–15 μg/L, which commonly defines ID in younger adults (Lipschitz et al., 1974; Ali et al., 1978; Carmel, 2008), has been claimed as too stringent in elderly patients. Indeed, in these subjects true ID often occurs at higher ferritin values, since ferritin *per se* raises with aging (Casale et al., 1981), and is an acute-phase reactant that increase during inflammation, infection, malignancy, and other illnesses common in older people. In a study carried out in hospitalized older patients, a serum ferritin level <50 μg/L resulted more reliable in predicting ID than other traditional cut-off values (Joosten et al., 1991). The low sensitivity of traditional iron biomarkers is demonstrated also by the fact that elderly anemic patients sometimes respond to iron supplementation even if their iron indices at baseline are not abnormal (Price et al., 2011).

Soluble transferrin receptor (sTfR), derived from proteolysis of the membrane transferrin receptor (TR), reflects erythropoietic activity and inversely correlates with the amount of iron available for erythropoiesis. In the past, some evidence supported sTfR measurement as a novel marker of ID in older people, considering that its levels do not increase with age and are not affected by the presence of inflammation (Mast et al., 1998). In particular, the serum sTfR divided by the log of the ferritin (sTfR-ferritin index) was found useful in classifying patients with ACD and concomitant IDA (Punnonen et al., 1997; Rimon et al., 2002). However, currently the lack of standardized reagents for the sTfR assay complicates interpretation of the sTfR-ferritin index in different studies, and limits its use in clinical practice (Pfeiffer et al., 2007).

In the last decade, hepcidin has been suggested as a promising diagnostic marker for iron-related disorders (Goodnough et al., 2010; Kroot et al., 2011). In IDA serum and urinary hepcidin levels are typically reduced and frequently undetectable by currently available assays (Bozzini et al., 2008; Ganz et al., 2008; Castagna et al., 2010). Hepcidin suppression appears also a sensitive indicator of ID without anemia, since decreased levels have been observed prior to a detectable decrease in Hb or hematocrit (Ganz et al., 2008; Pasricha et al., 2011). As mentioned above, hepcidin is induced by inflammatory cytokines and contributes to the pathogenesis of the so-called anemia of chronic disease (ACD), which is characterized by impaired iron utilization, along with inadequate EPO production, and cytokine-induced inhibition of erythroid precursors (Weiss and Goodnough, 2005). The opposite trend of hepcidin in IDA versus ACD has theoretically the potential to differentiate these conditions, both highly prevalent in elderly, and not infrequently coexisting. Of note, preclinical studies have shown that concomitant ID tends to blunt the hepcidin response to pro-inflammatory cytokines (Theurl et al., 2009; Darshan et al., 2010), suggesting the possibility to distinguish in the individual anemic patient the presence of IDA or mixed IDA/ACD (both with low to undetectable hepcidin levels) from ACD alone (with high hepcidin levels). Preliminary data in patients with rheumatoid arthritis (van Santen et al., 2011) or inflammatory bowel disease (Bergamaschi et al., 2013) are consistent with this possibility, but larger data are required for confirmation, particularly in elderly subjects where the distinction between IDA and ACD is expected to be particularly challenging.

## TREATMENT OF IDA IN ELDERLY

Currently, no specific guidelines exist for the management of anemia in the elderly. A recent review recommends that iron status should be checked at first in every elderly patient (Goodnough and Schrier, 2014). Once IDA is clearly ascertained or deemed likely (because of ambiguous results of iron markers as discussed above) a therapeutic trial with oral iron should be prescribed, with the aim of correcting both anemia and iron stores. This first-line approach, preferably using divalent compounds like ferrous sulfate or gluconate because of their superior bioavailability (Clark, 2009), is usually considered to be safer for the patient and with a better cost-effective ratio as compared to parenteral iron administration.

In general, Hb levels are expected to rise by approximately 1–2 g/dL every couple of weeks after starting oral iron therapy (Clark, 2008), which should be continued for 3 months after correction of anemia to replenish iron stores. The time needed may be even longer in elderly patients, because of slower bone marrow response. This translates in poor adherence, particularly when concomitant multimorbidity requires the assumption of a huge number of pills per day. Moreover, oral iron supplementation is often poorly tolerated in elderly patients, particularly because abdominal discomfort, as well as poorly absorbed because of the relative high prevalence of malabsorptive conditions (see above).

Thus, intravenous (IV) iron replacement is often required in elderly patients with IDA (Silverstein and Rodgers, 2004; Clark, 2009; Pasricha et al., 2010). Most IV iron formulations are generally effective, well tolerated, and with a lower incidence of serious adverse reaction (e.g., anaphylaxis) than commonly thought by many clinicians (Fishbane, 2003; Auerbach and Ballard, 2010; Auerbach et al., 2013a). Until a few years ago, the most widely used formulations in Europe were iron gluconate or iron sucrose, which are relatively unstable compounds with limited maximal doses per single infusion, i.e., 125 and 200 mg, respectively. Since the mean total iron dose generally required to correct anemia and restore iron is 1000–1500 mg, multiple hospital accesses for repeated infusions are needed. In elderly patients with limited autonomy, this not only increases direct and indirect social costs, but also can substantially hamper the feasibility of the treatment.

Recently, the pharmaceutical industry has made significant progresses through the production of more stable iron compounds that can be safely administered at high doses per single infusion, i.e., 1000–1500 mg, thus allowing a single treatment episode (for a review see Auerbach et al., 2013a). These includes low molecular weight iron dextran (Auerbach et al., 2011), ferumoxytol (Auerbach et al., 2013b), iron isomaltoside (Wikstrom et al., 2011), and ferric carboxymaltose (Evstatiev et al., 2011; Onken et al., 2014). These preparations significantly simplify the IV iron therapy, an effect that is expected to be particularly useful in elderly patients with IDA. However, specific trials in this setting are needed to confirm these exciting promises. Moreover, IV iron has theoretically the potential to generate oxidative stress and inflammatory cytokine production, and to increase susceptibility to certain infections, so that its long-term effects also need further future studies (Auerbach and Ballard, 2010; Goodnough et al., 2010).

**FIGURE 3:**
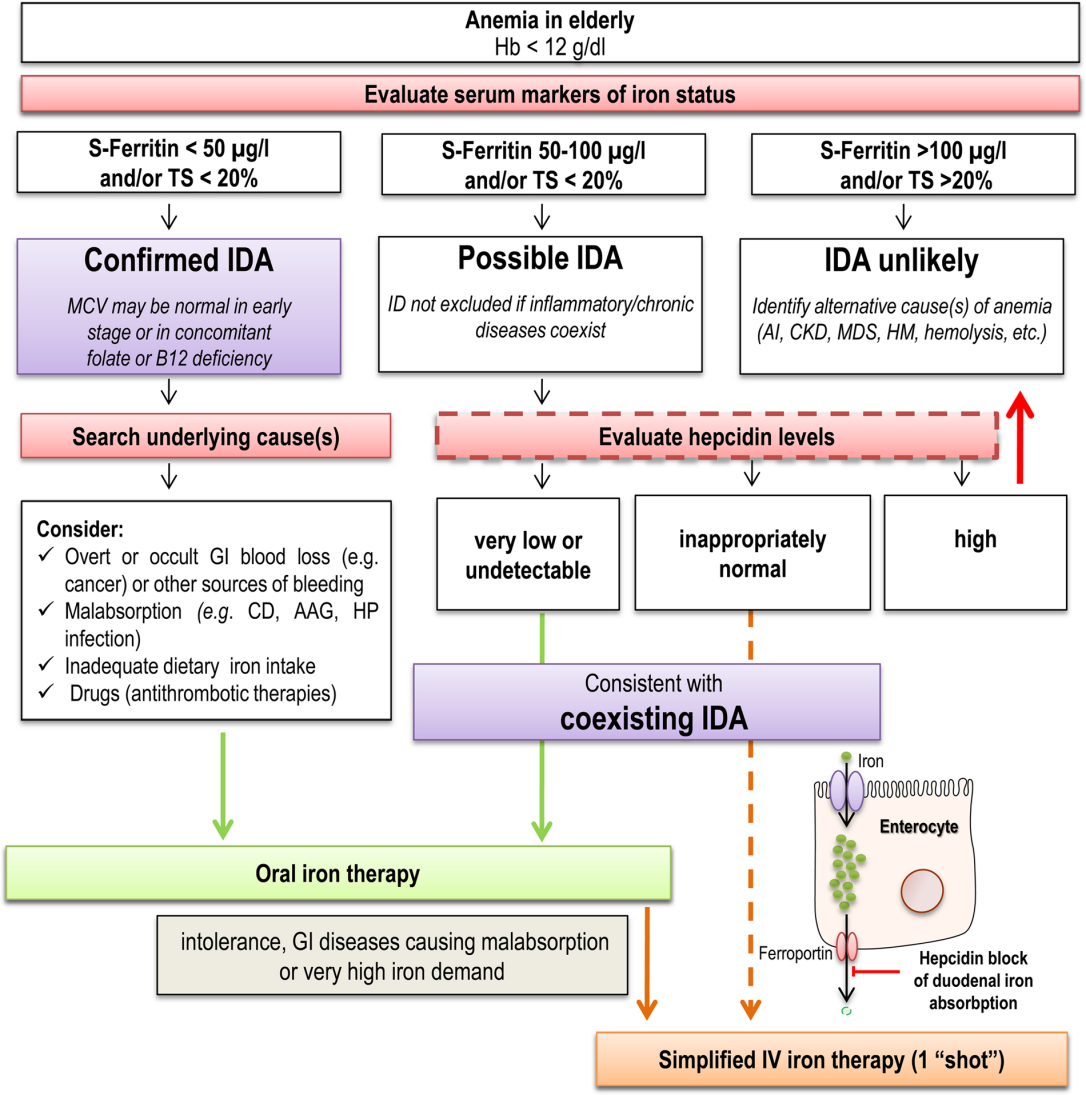
**Possible algorithm for the diagnosis and treatment of IDA in elderly in a near future scenario**. The proposed algorithm is based on most recent pathophysiological and therapeutic advances in the field. Dotted lines for evaluation of hepcidin levels indicate that the available data suggesting this approach (Bregman et al., 2013) need confirmation by future studies. Similarly, before this approach enters the clinical practice, a better standardization of hepcidin assays is needed. AAG, autoimmune atrophic gastritis; AI, anemia of inflammation; CD, celiac disease; CKD, chronic kidney disease; GI, gastrointestinal; Hb, hemoglobin; HM, hematologic malignancies; HP, Helicobacter pylori; IDA, iron deficiency anemia; MDS, myelodysplastic syndrome; TS, transferrin saturation.

Since hepcidin inhibits duodenal iron absorption, it has been suggested that measuring hormone levels may help to determine *a priori* the best way of iron administration, oral, or IV, in a given individual. Indeed, in a recent retrospective study, hepcidin levels were proven useful in identifying IDA patients who did not respond to oral iron supplementation (Bregman et al., 2013). Of note, the cut-off value of serum hepcidin that discriminated between responders (R) and not responders (NR) to oral iron was not particularly high (20 ng/ml), and within the “normal” range for the method employed (Bregman et al., 2013). The positive predictive value of NR for hepcidin levels >20 ng/ml was 81.4%. On the contrary, the majority of R patients had very low to completely suppressed (undetectable) hepcidin levels, as is the general rule for IDA patients (see above). If these data will be confirmed by further studies, hepcidin assay could actually help to individualize iron therapy, by avoiding waste of time with a poorly tolerated oral therapy when hormone levels are high or “pseudonormal,” particularly in elderly patients. In a near future scenario, a possible algorithm for the diagnosis and treatment of IDA in elderly, based on most recent pathophysiological and therapeutic advances in the field, is depicted in **Figure 3**.

## CONCLUSION

Iron deficiency is a major cause of anemia in elderly patients, and should be always searched as first diagnostic step. IDA in elderly is not infrequently multifactorial and difficult to diagnose since traditional biochemical markers of iron status are relatively ambiguous in this age group. Reasoning in terms of multimorbidity is central for a correct approach to IDA in elderly. Being GI blood loss the major cause of IDA in elderly, a correct identification of the bleeding source can be lifesaving, particularly when an occult malignancy is underlying. The discovery of hepcidin has revolutionized our understanding of iron metabolism, and measuring hormone levels may help both in diagnosis and in selecting the best treatment option, particularly as the IV therapy is becoming simpler than ever with the availability of new iron compounds.

## AUTHOR CONTRIBUTIONS

Fabiana Busti and Natascia Campostrini co-wrote the paper. Nicola Martinelli analyzed data. Domenico Girelli designed the work and co-wrote the paper. All authors have approved the final manuscript.

## Conflict of Interest Statement

The authors declare that the research was conducted in the absence of any commercial or financial relationships that could be construed as a potential conflict of interest.
